# Novel *MTND1* mutations cause isolated exercise intolerance, complex I deficiency and increased assembly factor expression

**DOI:** 10.1042/CS20140705

**Published:** 2015-03-27

**Authors:** Grainne S. Gorman, Emma L. Blakely, Hue-Tran Hornig-Do, Helen A.L. Tuppen, Laura C. Greaves, Langping He, Angela Baker, Gavin Falkous, Jane Newman, Michael I. Trenell, Bryan Lecky, Richard K. Petty, Doug M. Turnbull, Robert McFarland, Robert W. Taylor

**Affiliations:** * Wellcome Trust Centre for Mitochondrial Research, Institute of Neuroscience, Newcastle University, Newcastle upon Tyne NE2 4HH, U.K.; † Movelab, Institute of Cellular Medicine, Newcastle University, Newcastle upon Tyne NE2 4HH, U.K.; ‡ The Walton Centre NHS Foundation Trust, Liverpool L9 7LJ, U.K.; § Department of Neurology, Southern General Hospital, Glasgow G51 4TF, U.K.

**Keywords:** assembly factor, complex I, exercise intolerance, mitochondrial, *MTND1*

## Abstract

We describe the clinical, biochemical and molecular characterization of two adults with progressive exercise intolerance and severe isolated mitochondrial complex I (CI) deficiency due to novel *MTND1* mutations. We demonstrate compensatory CI assembly factor up-regulation probably partially rescuing the clinical phenotype.

## Abstract (precis)

Complex I (CI) is the largest of the five multi-subunit complexes constituting the human oxidative phosphorylation (OXPHOS) system. Seven of its catalytic core subunits are encoded by mitochondrial DNA (ND (NADH dehydrogenase)1–6, ND4L (NADH dehydrogenase subunit 4L)), with mutations in all seven having been reported in association with isolated CI deficiency. We investigated two unrelated adult patients presenting with marked exercise intolerance, persistent lactic acidaemia and severe muscle-restricted isolated CI deficiency associated with sub-sarcolemmal mitochondrial accumulation. Screening of the mitochondrial genome detected novel mutations in the *MTND1* (NADH dehydrogenase subunit 1) gene, encoding subunit of CI [Patient 1, m.3365T>C predicting p.(Leu20Pro); Patient 2, m.4175G>A predicting p.(Trp290*)] at high levels of mitochondrial DNA heteroplasmy in skeletal muscle. We evaluated the effect of these novel *MTND1* mutations on complex assembly showing that CI assembly, although markedly reduced, was viable in the absence of detectable ND1 signal. Real-time PCR and Western blotting showed overexpression of different CI assembly factor transcripts and proteins in patient tissue. Together, our data indicate that the mechanism underlying the expression of the biochemical defect may involve a compensatory response to the novel *MTND1* gene mutations, promoting assembly factor up-regulation and stabilization of respiratory chain super-complexes, resulting in partial rescue of the clinical phenotype.

## CLINICAL PERSPECTIVES

• We sought to assess *in vivo* and *vitro* mitochondrial function and evaluate the molecular mechanisms underlying a purely muscular phenotype in two adults whose clinical pictures were dominated by progressive exercise intolerance, lactic acidaemia and severe isolated mitochondrial CI deficiency in muscle.
• We describe the detailed clinical, physiological, biochemical and molecular characterization of these patients who we show to harbour novel heteroplasmic mutations in the mtDNA-encoded *MTND1* gene.
• We demonstrate for the first time that mitochondrial super-complex reorganization occurs as a response to a compensatory mechanism to partially extricate the clinical phenotype involving up-regulation of CI assembly factors, a phenomenon not previously reported with *MTND1* mutations.

## INTRODUCTION

Isolated deficiencies of human complex I (CI) often cause severe early-onset progressive disease, but the spectrum of disorders associated with this biochemical abnormality is expanding. In particular, mutations in the ND1 subunit of CI [MIM #252010] are associated with Leigh syndrome [MIM #256000]; cardiomyopathy; epilepsy; encephalopathy; mitochondrial encephalomyopathy, lactic acidosis and stroke-like episodes (MELAS) [MIM #54000]; Leber hereditary optic neuropathy (LHON) [MIM #535000] and an overlap syndrome comprising clinical features of both LHON and MELAS [1–7]. Exercise intolerance is a common symptom of mitochondrial disorders that can occur in isolation or as part of a multi-system disorder and has been associated with mutations in many genes encoding subunits of various complexes [8–11]. However, the relationship between the pathogenic mtDNA mutation and the biochemical and phenotypic expression of the defect remains poorly understood.

In the present paper, we describe two unrelated adult patients with severe isolated CI deficiency in skeletal muscle, progressive exercise intolerance, myopathy (without cardiomyopathy) and persistent lactic acidaemia. Both patients harbour novel heteroplasmic *MTND1* (NADH dehydrogenase subunit 1) gene [MIM #516000] mutations. We have characterized VO_2_ (oxygen uptake) kinetics during graded aerobic exercise, assessed *in vivo* mitochondrial function using phosphorus MR spectroscopy and evaluated the molecular mechanisms underlying this purely muscular phenotype to understand the impact of both mutations on CI biogenesis.

## MATERIALS AND METHODS

### Study approval

Local study approval was granted (NRES Committee North East- Newcastle & North Tyneside 1) and written informed consent from both patients was obtained prior to study inclusion. All clinical investigations were evaluated according to the Declaration of Helsinki.

### Subjects

Patient 1 presented at age 16 years with mild exercise intolerance and prominent fatigue following a viral illness. She was diag-nosed with chronic fatigue syndrome. At age 25 years she presented with progressive exertion-related dyspnoea and palpitations and was provisionally diagnosed with asthma. By 28 years of age, muscle weakness and fatigue with exercise-induced headache, vomiting, cardiac palpitations and syncope were prominent and a metabolic acidosis with elevated serum lactate was detected (Table 1). At this stage a neuromuscular opinion was sought. The clinical picture has progressed rapidly over the last 2 years with exercise tolerance reduced to less than 50 m. She has developed alcohol intolerance and postural orthostatic tachycardia syndrome (POTS). Patient 2 presented at age 22 years of age to a neurologist with life long indolent exertion-related muscle weakness and pain, dyspnoea, cardiac palpitations and syncope. There was no family history of muscle disease or parental consanguinity in either case.

**Table 1:** Peak exercise parameters, ^31^P-MRS examination on calf evaluating *in vivo* mitochondrial function and assessment of respiratory chain complex activities in skeletal muscle homogenates BPM, beats per min; DCPIP, 2,6-dichlorophenol-indophenol; n/a, not available; t1/2 PCR (s), half time for PCR recovery from end exercise to baseline concentrations; VO_2_, oxygen uptake. Enzyme activities are expressed as nmol of NADH oxidized/min per unit of citrate synthase (CS) for CI, nmol of DCPIP reduced/min per unit of CS for CII (succinate:ubiquinone-1 reductase) and the apparent first-order rate constant/s per unit of CS for CIII and CIV (×10^3^).

Parameter	Patient 1	Patient 2	Reference data (means ± S.D.)
CPET			
Peak exercise data	Absolute value (% of predicted)	Absolute value (% of predicted)	n/a
Heart rate (BPM)	172 (89)	180 (92)	n/a
VO_2_ (ml/min)	0.575 (27)	0.638 (22)	n/a
VO_2_ (ml/min per kg)	8 (27)	14 (22)	n/a
VO_2_ (% of predicted VO_2_)	27	22	n/a
Power (W)	35 (21)	53 (27)	n/a
AT			
ATVO_2_ (ml/min per kg)	6	10	n/a
ATVO_2_ (% of predicted peak VO_2_)	20	16	n/a
ATVO_2_ (% of recorded peak VO_2_)	75	74	n/a
			
^31^P-MRS			[14]
End exercise data			
t1/2 PCr	52.5	n/a	29.2 ± 3.1
t1/2 ADP	31.4	n/a	20 ± 1
			
Respiratory chain complex activities	Biopsy 1 Biopsy 2	Biopsy	Controls (*n*=25)
CI/CS	0.018 0.009	0.001	0.104 ± 0.036
CII/CS	0.092 0.127	0.070	0.145 ± 0.047
CIII/CS	0.673 1.790	0.634	0.554 ± 0.345
CIV/CS	0.834 1.394	1.216	1.124 ± 0.511

### Cardiopulmonary exercise testing

Cardiopulmonary exercise testing (CPET) was performed in both subjects as described elsewhere [12]. Anaerobic threshold (AT) was determined using the V-slope method, as previously described [13].

### Phosphorus magnetic resonance spectroscopy

Muscle phosphorus magnetic resonance spectroscopy (^31^P-MRS) was performed on the left calf, as detailed previously, in Patient 1 to evaluate *in vivo* mitochondrial function relative to an age matched reference group [14].

### Histochemical and biochemical analyses

Standard histological [H&E (haematoxylin and eosin), modified Gomori trichrome] and histochemical analyses of skeletal muscle biopsies were performed on fresh-frozen skeletal muscle sections (10 μm) [15]. Mitochondrial respiratory chain complex activities were determined in skeletal muscle homogenates and expressed relative to the activity of the matrix marker enzyme citrate synthase [16].

### Molecular genetics

Total DNA was extracted from available tissues including muscle, blood, buccal epithelia, urinary sediments, cultured myoblasts and fibroblasts. Muscle mtDNA rearrangements were investigated by long-range PCR strategies [17]. Direct sequencing of the entire mtDNA genome was undertaken [18]; alignment and variant calling were performed using SeqScape software (v2.1.1, Applied Biosystems) comparing changes to the GenBank reference sequence for human mtDNA (accession number NC_012920.1).

### Assessment of mtDNA mutation load by quantitative pyrosequencing

Heteroplasmic levels of the m.3365T>C and m.4175G>A mutations were determined in homogenate tissue and individual laser-microcaptured COX-positive and COX-positive ragged-red fibres (RRFs) by quantitative pyrosequencing. Quantification of the heteroplasmy level of each variant was achieved using Pyromark Q24 software [19].

### Mitochondrial respiratory chain complex subunit immunohistochemistry

Complex subunit immunohistochemistry was carried out on frozen tissue sections as described previously [20]. Primary antibodies and their dilutions used were: CI ND1, 1:200 (gift from Dr Anne Lombes), CI NDUFB8 1:50, CII SDHA (succinate dehydrogenase complex subunit A; 70 kDa flavoprotein subunit) 1:1000, CIII UQCRC2 (ubiquinone: cytochrome *c* reductase core protein 2) (Core 2) 1:1000, CIV MTCO1 (mitochondrial cytochrome *c* oxidase subunit 1) 1:1000, CIV MTCO4, 1:1000, Porin, 1:1000; all commercially available antibodies were purchased from Abcam. Primary antibodies were detected using a polymer detection system (Menarini Diagnostics) as per the manufacturer's instructions and visualized with 3,3′-diaminobenzidine (DAB; Sigma).

### BN/PAGE and SDS/PAGE

For BN (blue native)/PAGE, frozen skeletal muscle (10–50 mg) was homogenized and solubilized with 2% dodecylmaltoside (individual complexes and smaller super-complexes) or 4% digitonin (super-complexes) [21] and electrophoresed through precast 4–16% (dodecylmaltoside) or 3–13% gels (Invitrogen). Activities of CI, CII and CIV were estimated by in-gel assays as described in [22]. For 2D analysis, 1D lanes were incubated for 15 min in 1% SDS and 1% β-mercaptoethanol and separated in a 16% Tricine/SDS gel (BN-SDS/PAGE). For SDS/PAGE, proteins were extracted from enriched membranes (muscle) and separated by standard SDS/PAGE procedures. Proteins were transferred onto PVDF membranes (GE Healthcare) and processed for immunoblotting. Primary antibodies used were: NDUFB8 [NADH dehydrogenase (ubiquinone) 1β sub-complex 8], NDUFA9 [NADH dehydrogenase (ubiquinone) 1α sub-complex 9], Core2 (complex III Core protein 2), COXI, COXII, SDHA, CV subunits, TOM20 (translocase of outer mitochondrial membrane 20 kDa subunit) and porin (MitoSciences); AFG3L2 (AFG3-like AAA ATPase 2; Proteintech); LonP1 (Lon peptidase 1) and NDUFAF3 [NADH dehydrogenase (ubiquinone) complex I assembly factor; Sigma Life Science]; HSP60 (heat shock protein 60; BD Biosciences); NDUFAF1 and NDUFAF2 (gifts from Professor Leo Nijtmans) and ND1 (gift from Dr A. Lombes). Antibodies were detected with appropriate horseradish peroxidase (HRP)-conjugated immunoglobulins (Dako) and chemiluminescence detection reagents (GE Healthcare).

### Quantitative real-time PCR

Total RNA from control (*n*=4) and patient muscle was extracted using TRIrizol Reagent (Life Technologies) and reverse transcribed (0.5–1 μg) with random hexamers (Superscript III First Strand Synthesis System, Life Technologies). Levels of mRNA expression of *NDUFAF1* [MIM #606934], *NDUFAF2* [MIM #609653], *NDUFAF3* [MIM #612911] and *ACTB* (β-actin) [MIM #102630] were assessed in triplicate by real-time PCR using commercially-available TaqMan assays (assay IDs Hs00211245_m1, Hs02380072_u1, Hs00404252_g1 and Hs01060665_g1 respectively; Life Technologies). After normalization to *ACTB*, average expression levels of the CI assembly factor genes in patients were reported relative to control values, with differences assessed using a two-tailed Student's *t* test.

## RESULTS

### Clinical features

#### Patient 1

Patient 1 was of normal intelligence (Wechsler test of adult reading: thirty-seventh percentile), normal stature (1.7 m) and body habitus (68.7 kg). She had no ptosis and eye movements were normal. She did not have significant demonstrable clinical weakness at rest [Medical Research Council (MRC) 5/5]; however, subtle upper and lower limb proximal weakness (MRC 4+/5) was evident following minimal exertion (marching in place ten times). She also displayed marked evidence of increased ventilator rate (>30 breaths/min), sinus tachycardia (>120 beats/min) and bluish-hue discolouration to her digits. There was no evidence of a dysautonomia and no cardiac or respiratory muscle involvement. There was a persistent metabolic acidosis (serum bicarbonate 9–19 mmol/l; normal range 22–29 mmol/l), a high random serum lactate concentration [7.2–8.9 mmol/l; normal range < 1.7 mmol/l), markedly increasing post exertion up to 15.5 mmol/l and normal total creatine kinase activity (52–76 units/l; normal range <200 units/l). At the age of 27 years, there was electrophysiological evidence of a significant proximal myopathy with no findings of neuropathy. Conventional echocardiogram and cardiac MRI revealed no cardiac muscle abnormalities. She was treated with riboflavin (50 mg three times a day), high dose co-enzyme Q_10_ (500 mg/day) and L-carnitine supplements with minimal symptomatic or objective improvement. Current treatment strategies include energy conservation with the use of a wheelchair and pharmacological management with ivabradine for POTS.

#### Patient 2

Patient 2 was of normal height (1.68 m) but thin body habitus (47 kg). He had subtle unilateral ptosis and eye movements were normal. His skeletal muscles were generally poorly developed but he did not have significant demonstrable clinical weakness at rest (MRC 5/5); however, subtle upper and lower limb proximal weakness (MRC 4+/5) was evident following moderate exertion (marching in place 20 times). There was no evidence of cardiac or respiratory muscle involvement. There was a persistently high resting serum lactate concentration (4.1–6.8 mmol/l; normal range <1.7 mmol/l), increasing post exertion [15.4 mmol/l; cycling for 5 min; peak power 53W; peak oxygen consumption (VO_2_ peak) 14 ml/min per kg; AT 22% predicted VO_2_ peak] and raised total creatine kinase activity (351–486 units/l; normal range < 320 units/l). Electrophysiological changes at the age of 11 years were compatible with a myopathy with no findings of neuropathy.

### CPET

VO_2_ peak, peak work capacity (power; W), AT and absolute AT (expressed as a percentage of predicted peak VO_2_; ATVO_2_), were markedly decreased in both subjects (Table 1). Plasma lactate concentrations were concomitantly raised during CPET (Patient 1: resting lactate: 4.4 mmol/l, peak lactate: 13.2 mmol/l; Patient 2: resting lactate: 6.8 mmol/l, peak lactate: 15.4 mmol/l), with slow lactate decay time to baseline (Patient 1: 60 min, Patient 2: 40 min).

### ^31^P-MRS shows significant mitochondrial energy defects associated with isolated CI deficiency

^31^P-MRS examination showed that the ability of Patient 1’s muscle to reduce phosphocreatine (PCr) and accumulate ADP during exercise was normal. However, in recovery from muscle contraction, the main indicators of mitochondrial function demonstrated a severe impairment in oxidative metabolism (Table 1).

### Characterization of isolated complex I deficiency in skeletal muscle biopsies

**Figure 1:**
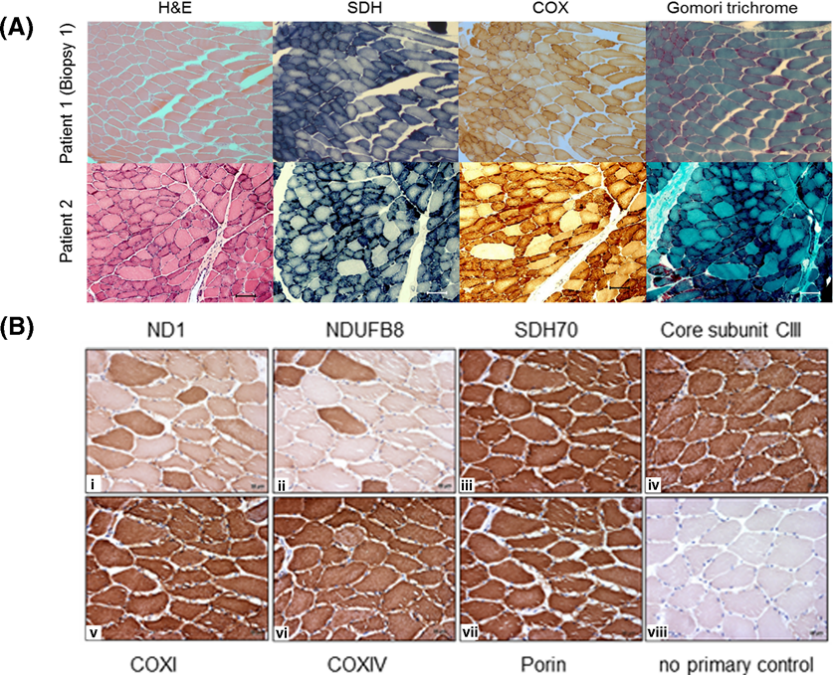
(**A**) Histological and histochemical assessment of patient muscle biopsies and (**B**) immunohistochemical analyses of muscle mitochondrial respiratory chain components in Patient 1 (**A**) Serial cryosections from both patients were assessed histologically (H&E and modified Gomori trichrome) and histochemically for oxidative enzyme activities including SDH and COX. Histological analysis of Patient 2’s biopsy revealed moderate variation in fibre size with abundant, slightly hypotrophic, fibre clusters showing sub-sarcolemmal and granular sarcoplasmic basophilia and occasional fibres with internal nuclei. Both the histochemical reaction and the modified Gomori trichrome staining confirmed sub-sarcolemmal mitochondrial accumulation typical of RRF ‘ragged-red’ fibres in both subjects. (**B**) i, CI ND1 subunit; ii, CI NDUFB8 subunit; iii, CII 70 kDa flavoprotein subunit; iv, CIII core protein 2; v, CIV subunit 1; vi, CIV subunit 4; viii, porin; viii, no primary antibody control. Immunocytochemical studies confirm very low expression of both CI-encoded subunits (NDUFB8 and ND1), whereas there was normal immunoreactivity to the structural components of all other mitochondrial respiratory chain complexes.

Oxidative enzyme reactions [succinate dehydrogenase (SDH) and cytochrome *c* oxidase (COX)] revealed numerous fibres with increased activity at the fibre periphery, confirmed by modified Gomori trichrome staining which showed sub-sarcolemmal accumulations typical of ‘ragged-red’ changes affecting >25% of all fibres in both patients (Figure 1A). Mitochondrial respiratory chain enzyme studies revealed isolated CI deficiency in skeletal muscle from both patients (Table 1), including a repeat muscle biopsy obtained from Patient 1 taken 2 years after her initial diagnostic biopsy. The biochemical defect in this patient was not expressed in cultured myoblasts (results not shown). Muscle analyses of Patient 1, shown in the present study, were performed on the first muscle biopsy. The activities of all other respiratory chain complexes were normal, confirming an isolated CI deficiency (Table 1). This was further confirmed by muscle immunohistochemistry in Patient 1 in which expression of both mtDNA-encoded (ND1) and nuclear-encoded (NDUFB8) CI structural components was markedly decreased in or absent from a vast number (>80%) of all muscle fibres (see Figures 1B,i and 1B,ii); protein components of CII, CIII and CIV showed normal expression (Figure 1B).

### Identification of novel pathogenic *MTND1* mutations

**Figure 2:**
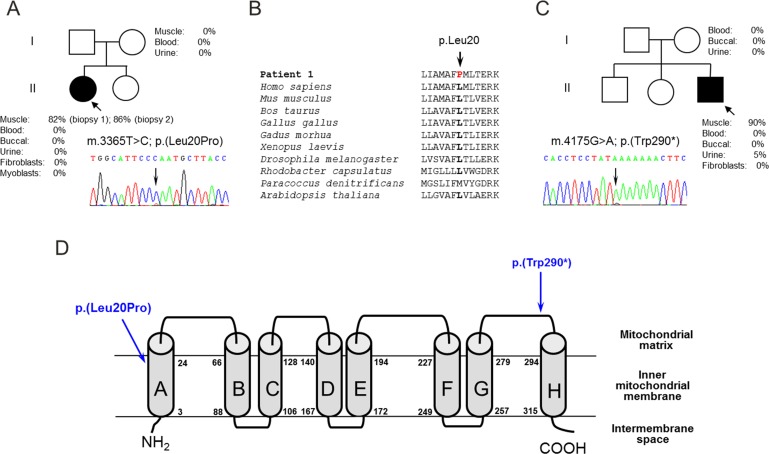
mtDNA sequencing reveals novel mutations in the *MTND1* gene (**A**) Sequencing of the mitochondrial genome identified an m.3365T>C mutation in Patient 1 predicting a p.(Leu20Pro) amino acid substitution. The mutation was present at high levels in patient muscle, but undetectable in all other tissue samples and maternal samples, consistent with a *de novo* mutation event. (**B**) Evolutionary sequence alignment of the relevant portion of the ND1 protein confirms p.Leu20 to be a highly conserved residue. (**C**) Sequencing of the mitochondrial genome identified an m.4175G>A mutation in Patient 2 predicting a p.(Trp290*) truncating event. This heteroplasmic mutation was present at high levels in patient muscle and at low levels in urinary sediment, but was also undetectable in available maternal DNA samples. (**D**) Predicted structure of human ND1 showing the eight transmembrane domains (http://sosui.proteome.bio.tuat.ac.jp) and the location of the p.(Leu20Pro) and p.(Trp209*) mutations, one within a transmembrane region and one within a conserved hydrophilic extra-membrane loop.

Having excluded mtDNA rearrangements, we determined the mtDNA sequence in muscle from both patients identifying novel candidate pathogenic *MTND1* mutations; Patient 1 harboured a novel m.3365T>C variant [predicting p.(Leu20Pro)] which was present at high levels of heteroplasmy (82% in biopsy 1; 86% in biopsy 2) in skeletal muscle, but undetectable in all other tissue samples tested (Figures 2A and 2B). Patient 2 was shown to harbour a novel m.4175G>A *MTND1* variant predicting p.(Trp290*) and premature truncation of the ND1 protein. Quantitative pyrosequencing showed that the m.4175G>A mutation was present at high levels of heteroplasmy in skeletal muscle (90% mutant load); at low levels (5% mutant load) in a urinary sediment-derived DNA sample, but undetectable in all other DNA samples (Figure 2C). Concurrent studies in the mothers of both patients failed to detect the respective mtDNA mutation suggesting *de novo* mutation events.

The m.3365T>C and m.4175G>A mutations were not previously reported on online databases of pathogenic mtDNA mutations [23,24] or within our own database of >980 human mtDNA sequences. Using quantitative pyrosequencing, we detected a trend towards higher levels of the m.3365T>C mutation in RRF (72.2±24.4%, *n*=10) than non-RRF (56.5±28.8%, *n*=10, *P*=0.2049, two-tailed Student's *t* test). For the m.4175G>A mutation, significantly higher levels of mutation were detected in COX-positive RRF (89.0±24.2% *n*=21) than in COX-positive non-RRF (39.6±44.2%, *n*=11) (*P*=0.0025, two-tailed Student's *t* test), confirming segregation of the m.4175A genotype with a histopathological abnormality.

### *MTND1* mutations are associated with loss of immunoreactive CI subunits and impaired CI assembly

**Figure 3:**
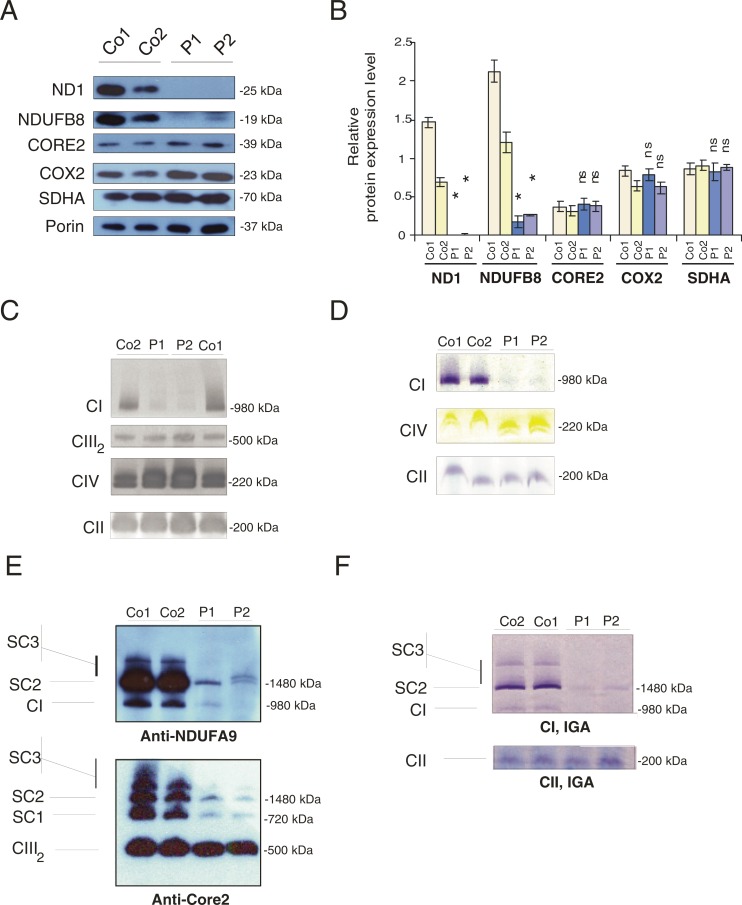
*MTND1* mutations lead to a loss of immunoreactive CI subunits and impair CI assembly (**A**) SDS/PAGE and Western blot analysis of muscle lysates (20 μg) from two controls (Co1, Co2) and two patients (P1, m.3365T>C mutation; P2, m.4175G>A mutation). Membranes were probed with antibodies directed against mitochondrial respiratory chain subunits and porin as a loading control. (**B**) Relative protein expression levels. Band intensity of indicated protein is normalized to band intensity of the loading control porin. Shown are mean values of three independent experiments ± S.D. *P<0.05 relative to controls; ns, not significant. (**C**) BN/PAGE of dodecyl maltoside (DDM)-solubilized muscle samples (75 μg) from two controls (Co1, Co2) and both patients with *MTND1* mutations (P1, P2) followed by Western blot analysis. Membranes were probed with anti-NDUFA9 for CI, SDHA for CII, CORE2 for CIII_2_ and COX4 for CIV. Blots are representative of two independent experiments. (**D**) In-gel enzyme activity (IGA) assay of respiratory chain complexes. BN/PAGE was performed on DDM-solubilized muscle samples (150 μg) from two controls and both patients. Enzyme activities for CI, CIV and CII were examined. (**E**) BN/PAGE of digitonin-solubilized muscle samples (100 μg) from two controls (Co1, Co2) and both patients (P1, P2) followed by Western blot analysis. Membranes were probed with anti-NDUFA9 and with anti-CORE2. SC1, CIII_2_ + CIV; SC2, CI + CIII_2_; SC3, CI + CIII_2_ + CIV. Blots are representative of two independent experiments. (**F**) IGA assay of mitochondrial super-complexes. BN/PAGE was performed on digitonin-solubilized muscle samples (200 μg) from two controls and the two patients with *MTND1* mutations (P1, P2). Enzyme activities for CI and CII were examined. SC1, CIII_2_ + CIV; SC2, CI + CIII_2_; SC3, CI + CIII_2_ + CIV.

The m.3365T>C; p.(Leu20Pro) mutation is located within the first transmembrane domain of the ND1 protein, whereas the m.4175G>A; p.(Trp290*) mutation occurs within the hydrophilic loop that faces the mitochondrial matrix between transmembrane domains G and H (Figure 2D). Western blot analysis of muscle from both patients failed to detect ND1 protein and identified markedly decreased levels of the nuclear-encoded CI subunit NDUFB8, whereas steady-state levels of other respiratory chain complex structural subunits were unaffected (Figures 3A and 3B). BN/PAGE analysis with subsequent Western blot analyses showed a fully assembled CI could be detected in both patients, albeit at very low levels, whereas steady-state levels of fully-assembled CII, CIII and CIV appeared unchanged (Figure 3C). This observation was accurately reflected in the in-gel activity assays which indicated some residual CI activity (Figure 3D), in agreement with immunocytochemical studies (Figure 1B).

BN/PAGE, followed by Western blot and in-gel-activity analyses of control muscle samples, showed a small amount of fully assembled CI in its monomeric form, whereas the majority of assembled complex was associated with other complexes building the SC2 (supercomplex 2) [CI + CIII_2_ (CIII dimer)] and SC3 (CI + CIII_2_ + CIV_(1–4)_) super-complexes (Figure 3E). Levels of fully assembled CI were strongly decreased in patient samples, with residual CI associated, in the most part, with CIII in the SC2 (CI+CIII_2_) super-complex (Patient 1) and with CIV in the SC3 (CI + CIII_2_ + CIV_(1–4)_) super-complex (Patient 2; Figures 3E and 3F).

### *MTND1* mutations stimulate an increase in CI assembly factor expression

**Figure 4:**
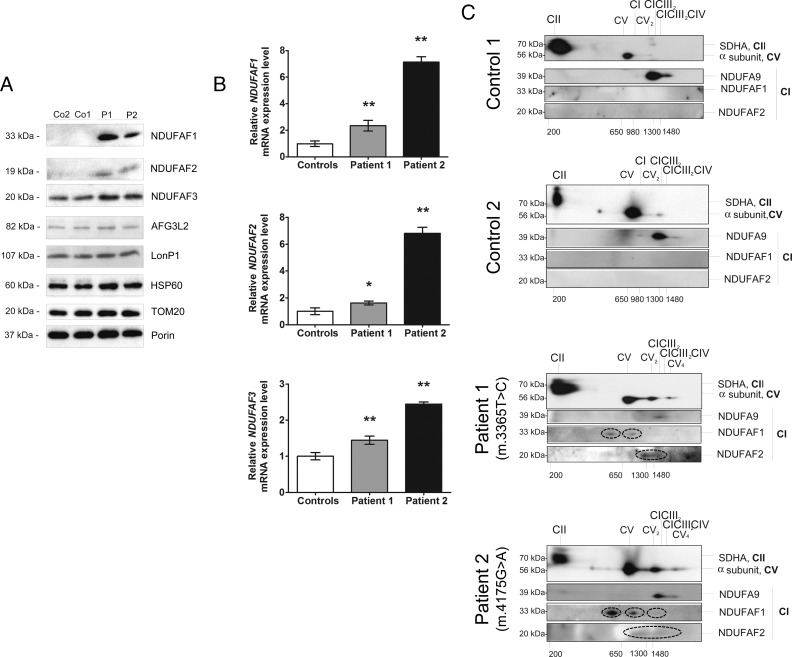
(**A** and **B**) *MTND1* mutations stimulate an increase in CI assembly factor expression and (**C**) 2D BN/PAGE analysis of muscle mitochondria identifies smaller CI sub-complexes associated with *MTND1* mutation (**A**) SDS/PAGE and Western blot analysis of muscle lysates (20 μg) from two controls (Co1, Co2) and both patients (P1, m.3365T>C mutation; P2, m.4175G>A mutation). Membranes were probed with antibodies directed against the CI assembly factors NDUFAF1, NDUFAF2 and NDUFAF3, the mitochondrial AAA protease AFG3L2, the LonP1 protease, the HSP60 and porin as a loading control. Blots are representative of two independent experiments. (**B**) Relative mRNA expression levels of three CI assembly factor genes. The mRNA expression levels of *NDUFAF1*, *NDUFAF2* and *NDUFAF3* were measured by real-time PCR in Patients 1 and 2 and controls (*n*=4) and normalized to *ACTB* mRNA levels. Average patient data from three replicate experiments are expressed relative to control values ± S.D. *P<0.015; **P<0.001 relative to controls. (**C**) Digitonin-solubilized mitochondrial complexes were separated by 1D BN/PAGE, followed by 2D Tricine-SDS/PAGE and Western blot analysis. Membranes were probed with SDHA for CII, α subunit for CV, NDUFA9 for CI and NDUFAF1 and NDUFAF2. SC1, CIII_2_ + CIV; SC2, CI + CIII_2_; SC3, CI + CIII_2_ + CIV. The smaller sub-complexes observed in both patients are highlighted by the ellipses; NDUFAF1 was associated with two CI intermediates of ∼500 and ∼800 kDa, whereas NDUFAF2 was detected within higher-molecular-mass associations corresponding to complexes of 800 and 1500 kDa in the muscle of Patients 1 and 2.

We evaluated the steady-state levels of different CI assembly factors including NDUFAF3 (early-stage factor), NDUFAF1 (involved in the middle stages) and NDUFAF2 (involved in the late stages of CI assembly) and mitochondrial proteases including AFG3L2, LonP1 and HSP60. We observed no changes in the expression levels of any proteases; however, steady-state levels of all three CI assembly factors were markedly increased in patient muscle samples suggestive of a compensatory response to the ND1 defect (Figure 4A). Confirmatory real-time PCR analy-ses showed increased *NDUFAF1* [MIM #606934], *NDUFAF2* [MIM #609653] and *NDUFAF3* [MIM #612911] transcript levels in patient muscle (Figure 4B).

BN-SDS/PAGE analysis of digitonin-solubilized control and patient muscle samples showed that the majority of NDUFA9 was associated with the SC2 (CI + CIII_2_) and SC3 (CI + CIII_2_ + CIV_(1–4)_) super-complexes in controls (Figure 4C). Both NDUFAF1 and NDUFAF2 were undetectable in control muscle. In the patient muscle samples, the majority of NDUFA9 was detected in association with the SC2 (CI + CIII_2_) and SC3 (CI + CIII_2_ + CIV_(1–4)_) super-complexes. However, smaller sub-complexes were also observed in both patients. We found that NDUFAF1 was associated with two complex I intermediates of ∼500 and ∼800 kDa, whereas NDUFAF2 was detected within higher-molecular-mass associations corresponding to complexes of 800 and 1500 kDa in muscle of both patients (Figure 4C). In the patient samples, we observed higher amounts of CV in higher supramolecular organizations (as dimer V_2_ and tetramer V_4_; Figure 4C), whereas the majority of mt-ATP synthase was found as monomers in controls.

## DISCUSSION

We describe novel sporadic *MTND1* mutations in two unrelated patients with isolated CI deficiency characterized clinically by marked exercise intolerance and *in vivo* mitochondrial dysfunction. The clinical phenotypes, severity of the biochemical defect and the relatively indolent nature of the disease are conspicuously similar in both patients. Neurological examination was essentially normal in the resting state; however, response to exercise in both cases was indicative of a mitochondrial myopathy. Both cases highlight the importance of serum lactate testing in cases of persistent unexplained exertional weakness or dyspnoea. Indeed, mitochondrial disorders are recognized as a common cause of referral for unexplained dyspnoea, with a period prevalence of up to 8.5% [25].

Several strands of evidence indicate that the *MTND1* mutations are pathogenic, causing the clinical phenotype. The m.3365T>C and m.4175G>A mutations are novel and absent from available databases of characterized mtDNA mutations, whereas both *MTND1* variants were heteroplasmic and segregate with disease in muscle and, for Patient 2, with the histopathological abnormality (COX-positive RRF). The undetectable steady-state levels of ND1 protein suggest that both *MTND1* mutations affect production and/or stability of the protein with dramatic effects on CI assembly and degradation of other CI subunits, including NDUFB8. Intriguingly, ND1 protein deficiency does not totally preclude assembly of the holocomplex, similar to that observed in a patient with progressive encephalopathy due to a *B17.2L* (*NDUFAF2*) [MIM #609653] null mutation [26].

Smaller CI subunits were also detectable with several intermediate complexes clearly seen in association with CI assembly factors. Accumulation of stalled intermediates of ∼500 kDa and ∼800 kDa has previously been reported in patients with various CI assembly pathway mutations who also exhibit reduced assembly of the holoenzyme complex [26–31]. Steady-state levels of the mitochondrial quality-control machinery including the AAA protease AFG3L2, LonP1 protease and HSP60 remained unchanged in association with up-regulation of assembly factor for intermediate-stage (NDUFAF1) and late-stage (NDUFAF2) CI assembly in patient muscle, suggesting a shift in this dynamic equilibrium state that favours assembly and stability of respiratory complex formation over degradation.

We speculate that mutation load and tissue-specific effects in CI formation and stability, mediated through assembly factor up-regulation, act as a compensatory response to the deleterious *MTND1* genetic defect, with an absence of secondary defects in CII, CIII and CIV further facilitating complex stabilization. Taken together, our findings all support assembly factor up-regulation in isolated CI deficiency as an adaptive process to stabilize the respiratory complexes, a phenomenon not previously reported with *MTND1* mutations [32] and that the pathogenic role of these mutations at a molecular level is dependent on the stage of CI assembly. Mitotic segregation has long been recognized to contribute to phenotypic variability that may explain the isolated muscle defects in these patients, although we concede that definitive exclusion of CNS involvement cannot be definitively made based on current evaluations.

In summary, we report two novel *MTND1* mutations that clinically manifest as isolated exercise intolerance and at a molecular level cause assembly factor up-regulation and sub-complex assembly acting to stabilize respiratory chain complexes and salvage assembly of the holoenzyme complex.

## Abbreviations

^31^P-MRS: phosphorus magnetic resonance spectroscopy
ACTB: β-actin
AFG3L2: AFG3-like AAA ATPase 2
AT: anaerobic threshold
BN: blue native
CI/CII/CIII/CIV/CV: complex I/II/III/IV/V
CIII_2_: CIII dimer
COX: cytochrome *c* oxidase
CPET: cardiopulmonary exercise testing
H&E: haematoxylin and eosin
HSP60: heat shock protein 60
LHON: Leber hereditary optic neuropathy
LonP1: Lon peptidase 1
MELAS: mitochondrial encephalomyopathy, lactic acidosis and stroke-like episodes
MRC: Medical Research Council
NDUFA9: NADH dehydrogenase (ubiquinone) 1α sub-complex 9
NDUFAF: NADH dehydrogenase (ubiquinone) complex I assembly factor
NDUFB8: NADH dehydrogenase (ubiquinone) 1β sub-complex 8
PCr: phosphocreatine
POTS: postural orthostatic tachycardia syndrome
RRF: ragged-red fibre
SDH: succinate dehydrogenase
SDHA: succinate dehydrogenase complex subunit A
VO_2_: peak, peak oxygen consumption
VO_2_: oxygen uptake
